# Passive protection against anthrax in mice with plasma derived from horses hyper-immunized against *Bacillus anthracis* Sterne strain

**DOI:** 10.7717/peerj.3907

**Published:** 2017-12-15

**Authors:** Marc Caldwell, Terri Hathcock, Kenny V. Brock

**Affiliations:** 1 Department of Pathobiology, Auburn University, Auburn, AL, United States of America; 2 Edward Via College of Osteopathic Medicine, Auburn University, Auburn, AL, United States of America

**Keywords:** Anthrax, Equine, Mouse lethal challenge, Passive immunotherapy, *B. anthracis* sterne strain

## Abstract

In this study, equine source polyclonal anti-*Bacillus anthracis* immunoglobulins were generated and utilized to demonstrate passive protection of mice in a lethal challenge assay. Four horses were hyper-immunized with *B. anthracis* Sterne strain for approximately one year. The geometric mean anti-PA titer in the horses at maximal response following immunization was 1:77,936 (Log2 mean titer 16.25, SEM ± 0.25 95% CI [15.5 –17.0]). The geometric mean neutralizing titer at maximal response was 1:128 (Log2 mean titer 7, SEM ± 0.0, 95% CI 7). Treatment with hyper-immune plasma or purified immunoglobulins was successful in passively protecting A/J mice from a lethal *B. anthracis* Sterne strain challenge. The treatment of mice with hyper-immune plasma at time 0 h and 24 h post-infection had no effect on survival, but did significantly increase mean time to death (*p* < 0.0001). Mice treated with purified immunoglobulins at time 0 h and 24 h post-infection in showed significant increase in survival rate (*p* < 0.001). Bacterial loads in lung, liver and spleen tissue were also assessed and were not significantly different in mice treated with hyper-immune plasma from placebo treated control mice. Mice treated with purified antibodies demonstrated mean colony forming units/gram tissue fourfold less than mice receiving placebo treatment (*p* < 0.0001). Immunotherapeutics harvested from horses immunized against *B. anthracis* Sterne strain represent a rapidly induced, inexpensive and effective expansion to the arsenal of treatments against anthrax.

## Introduction

Antibiotic therapy is the foundation for post-exposure prophylaxis and treatment of anthrax infection (Hendricks et al., 2014; Pillai et al., 2015). Removal of vegetative bacteria from circulation is necessary to curtail systemic spread and decrease morbidity and mortality (Bryskier, 2002; Weiss et al., 2011). In its vegetative state, *Bacillus anthracis* constitutively produces the anthrax exotoxins, lethal toxin and edema toxin, and these toxins continue to contribute to pathogenesis and ongoing illness beyond clearance of the live organisms (Moayeri et al., 2015). Neutralization of the three toxin protein subunits (lethal factor, LF, edema factor, EF, and protective antigen, PA) via immunization and administration of passive monoclonal and polyclonal immunotherapeutics improves the clinical outcomes of systemic forms of the disease (Hendricks et al., 2014; Huang et al., 2015). Efficacy studies of passive and active immunity indicate that PA is the most important target for neutralizing both lethal and edema toxins (Ivins et al., 1992; Turnbull et al., 1986; Turnbull et al., 1988). Protective antigen is the common subunit for each toxin and mediates cellular translocation of the effector molecules through the formation of intra-phagocytic pores (Pezard, Berche & Mock, 1991). Effective antibody interference with PA prevents the manifestation of the *B. anthracis* toxin pathogenesis (Albrecht et al., 2007; Karginov et al., 2004). In addition, neutralization of LF and EF have each been independently shown to improve survivability and reduce morbidity (Albrecht et al., 2007; Leysath et al., 2011; Pohl et al., 2013). A combination of these targets may provide the most robust protection, but to date targeting PA has been the focus of most therapeutic advances (Pohl et al., 2013; Staats et al., 2007).

There are currently two US Food and Drug Administration approved immunotherapeutics indicated for treatment of and prophylaxis against inhalational anthrax in adults and children in combination with appropriate antibiotics. Raxibacumab (GlaxoSmithKline, London, UK) is a human IgG1λ monoclonal antibody produced in a murine hybridoma expression system that specifically binds PA (US-FDA, 2013). The efficacy of raxibacumab has been evaluated in challenge studies utilizing toxin administration or fully virulent *B. anthracis* inoculation in rats, rabbits, dogs and non-human primates (Corey et al., 2013; Cui et al., 2005; Kummerfeldt, 2014; Mazumdar, 2009; Migone et al., 2015; Migone et al., 2009; Remy et al., 2015). Anthrax Immune Globulin Intravenous (AVIG, Anthrasil, Cangene Corporation, Winnipeg, Manitoba, Canada) is a human polyclonal antiserum derived from the plasma of persons immunized with anthrax vaccine adsorbed (AVA, BioThrax, Emergent BioSolutions Inc., Rockville, MD, USA; US-FDA, 2015). Its efficacy has been evaluated in rabbits and non-human primates in the treatment and prophylaxis of fully virulent *B. anthracis* challenges (Kammanadiminti et al., 2014; Mytle et al., 2013). Because AVA is an inactivated whole cell lysate of a fully toxigenic and encapsulated strain of *B. anthracis,* plasma derived from individuals immunized with this product contains multiple epitopic targets including all three toxins subunits as wells other capsular and non-capsular antigens (Wright et al., 2010).

The use of equine antisera for the treatment and prevention of human infectious disease pre-dates the antibiotic era (Von Stabsarzt, 1991). In 1901, Emil von Behring received the first Nobel Prize in Physiology and Medicine for developing polyclonal antibodies (pAbs) and using them as a treatment against diphtheria and tetanus (Von Behring, 2008). Shortly after an Italian protégé of von Behring, Achille Sclavo, began work on developing a donkey based antiserum against anthrax to stem the effect the disease was creating in Italy at the time (Ragazzi, 1954). The first report of successful treatment using Sclavo’s anti-serum appeared in the *British Medical Journal* in 1905 describing the treatment and recovery of a man with cutaneous anthrax (Mitchell, 1905). Reports of the observational success of anti- serum in the treatment of cutaneous and systemic anthrax continued up until 1954 (Hodgson, 1941; Canright, 1928; Santee, 1923; Sclavo, 1954). Equine-based hyperimmune products continue to be important therapeutic modalities for toxin based diseases such tetanus, botulism, and snake envenomation, as well as some viral disease such rabies (Arnon, 2007; CDC, 2010; CDC, 2011). Due to low manufacturing cost, simplified logistics, high antibody yield and low risk of transfusion infections, the hyperimmunization of horses against anthrax represents a compelling alternative compared to the costly manufacture of monoclonal antibodies and the limited supply of AVA vaccinated plasma donors. Developing nations with limited resources and biomedical infrastructure could utilize an equine-derived manufacturing process to gain access to these types of therapies. This study describes a proof-of-concept analysis whereby horses were immunized with the veterinary *B. anthracis* Sterne 34F2 strain spore vaccine to develop a polyclonal hyperimmune plasma. This plasma was assessed for its anti-PA IgG titer as well as lethal toxin neutralizing titer. Then the hyperimmune plasma and plasma-derived purified IgG immunoglobulins were assessed for efficacy in passively protecting A/J mice from a lethal intranasal (IN) and subcutaneous (SC) *B. anthracis* Sterne strain spore challenge.

## Materials and Methods

### Equine immunization

All procedures described within this study involving the use of animals were approved by Auburn University Institutional Animal Care and Use Committee (protocol #2012–2105). Four Percheron draft mares were immunized with 1.0 ml of *B. anthracis* Sterne strain spore vaccine (Anthrax Spore Vaccine, Colorado Serum Company, Denver, CO, USA) once monthly for a total of 12 immunizations over the course of approximately 302 days. Whole blood samples were collected at monthly intervals (12 total blood collections) for titer analysis. Samples were centrifuged at 5,000× g for 20 min, plasma separated, and stored at −80 °C until ready for analysis. Plasma used for negative controls were obtained from two un-immunized horses (control plasma, CON) as described for principal samples.

### Quantification of anti-PA IgG antibodies

A 96-well plate (Immulon 2 HB Southern Biological, Birmingham, AL, USA) was coated with 100 µl of rPA coating solution (1 µg/ml in PBS, List Biological Laboratories, Inc. Campbell, CA, USA). Plates were incubated overnight at 4 °C . Principal samples (1:500) were added in triplicate. Naïve control plasma samples (1:50) were also added in triplicate. Twofold dilutions were made across the plate, with 100 µl discarded from the last well. The plate was then incubated at 37 °C for 1 h. Goat anti-equine IgG antibody conjugated to HRP (Jackson Immunoresearch Lab, INC, West Grove, PA, USA) was added to each well and the plate was incubated again at 37 °C for 1 h. Finally, ABTS (Thermo Scientific, Rockford, IL, USA) was added, followed by a brief incubation and determination of optic density performed (405 and 550 nm, Bio-Tek Elx800 plate reader, Winooski, VT, USA). A reference plasma or positive standard was developed from a pool of vaccinated horses and was subjected to a series of assays in order to establish a titration curve. Briefly, eight rPA-coated microtiter plates were prepared. Following overnight incubation, approximately 200 µl of the reference plasma that had been diluted to 1:500 was added to the first well of three rows on each plate then two-fold serial dilutions carried out. In this way, each plate contained reference plasma samples in triplicate. A titration curve was drawn using individual absorbance values at each dilution point from the 24 assays relating the log10 experimental units/mL vs. log10 OD. Once the titration curve was established a series of experiments were conducted to establish the limit of detection. Two samples of naïve horse plasma were diluted 1:50 and subjected to the anti-PA IgG ELISA in triplicate in two microtiter plates, on each of the five days within two weeks. Fresh dilutions and rPA-coated plates were individually prepared and used daily. The limit of detection was estimated by interpolating the mean of 60 absorbance values, plus three standard deviations, in a titration curve of reference plasma relating log10 -assigned units/ml per well vs. log10 absorbance. The antilog of the interpolated value was subsequently corrected by the dilution of the sample (1:50). End point titers were calculated as the highest dilutions at which the OD values were two fold higher the interpolated limit of detection. The geometric mean for each sample was calculated from the estimated end-point titer from triplicate samples. Once established, the reference plasma was used as a positive standard along with each of the naïve horse plasma samples as negative reference standards in every assay thereafter. Table 1 lists the estimated geometric mean anti-PA IgG log titers.

**Table 1:** Reciprocal end point anti-protective antigen titers for horses immunized monthly with 1.0 ml Sterne strain *Bacillus anthracis* spore vaccine. Plasma from each horse was subjected to the anti-PA IgG indirect ELISA for each time point. Reciprocal titers were achieved from the geometric mean of three replicates of each sample.

	Mean Reciprocal Titers					
Days of Immunization	AX1	AX2	AX3	AX4	Naïve 1	Naïve 2
0	0	0	0	0	0	0
21	2,000	0	500	500	0	0
50	16,000	32,000	32,000	32,000	0	0
78	32,000	16,000	32,000	32,000	0	0
106	64,000	64,000	32,000	64,000	0	0
134	32,000	64,000	16,000	16,000	0	0
162	64,000	64,000	32,000	64,000	0	0
190	32,000	64,000	64,000	128,000	0	0
218	64,000	64,000	128,000	64,000	0	0
246	32,000	64,000	32,000	32,000	0	0
274	32,000	64,000	128,000	32,000	0	0
302	64,000	32,000	128,000	16,000	0	0

### Antibody mediated neutralization of lethal toxin

Lethal toxin neutralizing titers were determined by modifying a previously described assay for human and rabbit antisera (Hering et al., 2004). Mouse macrophage J774A.1 cells (ATCC TIB-67) were obtained and grown in high-glucose Dulbecco’s modified Eagle medium containing l-glutamine and supplemented with 10% fetal bovine serum and 1% penicillin-streptomycin (10,000 U/ml penicillin G sodium and 10,000 µg/ml streptomycin sulfate).

Two-fold dilutions of plasma (1:32) were prepared in supplemented DMEM. Recombinant protein solutions of rPA (1.0 µg/ml) and rLF (1.0 µg/ml) were added to the serum dilutions, and the mixtures were incubated for 10 min at 37 °C with shaking. Cells were diluted to 2 × 10^6^ cells/ml, then added to a sterile 96-well tissue culture plate and incubated for 2 h. The media was removed and replaced with 100 µl media containing prepared toxin-plasma mixtures and incubated for 4 h at 37 °C in 5% CO_2_. alamarBlue (80% solution in Hanks balanced salt solution; Trek Diagnostic Systems Inc., Westlake, OH, USA) was added at 10% of the well volume, and the cells were incubated for 20 h at 37 °C in 5% CO_2_. Absorbance at 570 nm (to detect oxidized alamarBlue) and 595 nm (to detect reduced alamarBlue) was measured using a Bio-Tek Elx800 plate reader. Cells lysed by the addition of 10 µl of Triton X-100 were used as negative controls. As stated above, end point titers were determined as the highest dilutions at which the OD values were two fold higher the interpolated limit of detection for the assay. Assays were performed in triplicate. The geometric mean for each sample was calculated from the estimated end-point titer from triplicate samples. Table 2 lists the estimated geometric mean lethal toxin neutralizing log titers.

**Table 2:** Reciprocal endpoint neutralizing titers for horses immunized with 1.0 ml Sterne strain *Bacillus anthracis* spore vaccine. J774A.1 cells were exposed to *B. anthracis* lethal toxin (1.0 µg/ml PA and 1.0 µg/ml LF) that had been pre-incubated with individual hyper-immune plasma samples. alamarBlue was added overnight and the amount of reduced alamarBlue was measured as an indicator of metabolic activity. The neutralization titer, defined as the highest dilution conveying 50% protection from the LF-mediated lysis, is plotted for each plasma sample at each time point. Each titer represents the geometric mean for three replicates.

	Mean reciprocal titers					
Days of immunization	AX1	AX2	AX3	AX4	Naïve 1	Naïve 2
0	0	0	0	0	0	0
21	32	32	32	32	0	0
50	128	128	128	128	0	0
78	128	128	128	128	0	0
106	128	128	128	128	0	0
134	128	128	128	128	0	0
162	128	128	128	128	0	0
190	128	128	128	128	0	0
218	128	128	128	128	0	0
246	128	128	128	128	0	0
274	128	128	128	128	0	0
302	128	128	256	128	0	0

### Passive protection assay

#### Purification and preparation of plasma and immunoglobulin treatments

Plasma treatments were prepared from four aliquots demonstrating the highest anti-PA IgG and neutralizing titer (1:128,000 and 1:128–1:256, respectively) and pooled immediately prior to administration. The placebo treatments were prepared from CON plasma and was handled in the same manner. Administration of 0.8 ml of each was chosen as a maximum safely administered volume.

To produce the purified immunoglobulin samples, aliquots corresponding to those plasma samples used as treatments above were subjected to polyclonal protein A columns for affinity purification (Pierce Protein A Columns, Pierce-Thermo Scientific, Rockford, IL, USA). Fractions with the highest protein content were pooled and stored at −80 °C until ready to use. Placebo treatments consisted of immunoglobulin fractions obtained from CON plasma that had been subjected to the same purification and preparation procedures.

#### Inoculum preparation

The challenge bacterium used was obtained from the veterinary vaccine *B. anthracis* Sterne strain 34F2 (Anthrax Spore Vaccine, Colorado Serum Company Denver, CO, USA). The procedure to determine the lethal dose 50 (LD_50_) of the *B. anthracis* Sterne strain spores in A/J mice was slightly modified from that described by Hewetson et al. (2008) Briefly, mice received a range of spores suspended in PBS from 10 to 1 × 10^6^ either intra-nasally (IN) or subcutaneously (SC). The estimated log_10_ of the IN LD_50_ from three independent studies was 3.5718 (upper and lower 95% CI: 4.1163 and 3.0351). For subsequent IN challenges an infectious dose of 4 LD_50_, or approximately 1.5 ×10^4^ spores was used. Additionally, the estimated log_10_ of the SC LD_50_ was 2.7383 (upper and lower 95% CI: 3.2232 and 2.1798). For subsequent SC challenges an infectious dose of approximately 2 × 10^3^ spores was used. Stock preparations of both challenge inoculums were verified for spore concentration immediately before and after challenge procedures were performed.

#### *B. anthracis* challenge

Female A/J strain mice were obtained from Jackson Laboratory (Bar Harbor, ME, USA) and were from 6–12 weeks age during these experiments. Mice were housed in ventilation controlled hepa-filtered biosafety cages with up to five animals per cage. The mice were divided into seven treatment groups for each route of challenge as described in  Tables S.1 and S.2. Mice were randomly allocated to the following treatment groups: **Control (CON)** mice treated with plasma collected from horses naïve against *B. anthracis* (*n* = 20 in the plasma experiment, *n* = 15 in the purified IgG experiment); **Non-infected Treatment Control** **(Non-Infected)** mice received immune plasma (*n* = 20) or concentrated IgG (*n* = 15) and sterile PBS as a sham IN and SC inoculation; **Immune plasma**, mice receiving immune plasma were treated at 0 h (**Tx 0 h**) and 24 h (**Tx 24 h)** following IN or SC challenge (*n* = 20 for each challenge group); and **Purified Immunoglobulins**, mice receiving immunoglobulins were treated at 0 h (**IgG 0 h**) and 24 h (**IgG 24 h**) following IN or SC challenge (*n* = 15 for each group). All treatments were administered once at 0.8 ml volume in the right rear quadrant of the peritoneal cavity while mice were restrained. Treatments administered at 0 h were treated once concurrently at the time of challenge, while the 24 h treated groups received a single treatment at 24 h post-challenge.

Intranasal challenges were carried out by first anesthetizing each mouse (xylazine/ketamine combination, 10 mg/kg and 100 mg/kg respectively, IP) and depositing 30 µl PBS containing approximately 1.5 × 10^4^ spores on the external nares. Mice were restrained until all the liquid was aspirated. Sham IN challenged mice were anesthetized and handled in an identical manner, however 30 µl of sterile PBS was deposited on the external nares. For SC infection, un-anaesthetized mice were injected with 100 µl PBS containing approximately 2 × 10^3^ spores under the skin of the shoulders. Sham SC challenged mice were handled in an identical manner but, 100 µl of sterile PBS was administered SC. Mice were observed twice daily for 7 days. As mice died, tissues were collected for determination of bacterial concentrations. At the end of the 7-day study period all surviving mice were humanely euthanized.

#### Tissue bacterial concentrations

Lungs, liver, and spleen were collected immediately following death of each mouse. The tissues were weighed and manually homogenized. Following homogenization, an equal weight by volume of sterile PBS was added to each tissue homogenate and 1 ml of the homogenate was serially diluted 1:10 in sterile PBS. Twenty µl of each dilution was plated onto BHI agar plates in triplicates and incubated at 37 °C for 24 h. Colony forming units were determined using the Miles and Misra method and calculations made to determine the CFUs/gram of tissue (Hedges, 2002).

### Statistical analysis

Geometric mean titers were calculated by logarithmic transformation and group means compared by repeated measures ANOVA. Probit analysis was used to calculate LD_50_ values. Survival rates were compared by Fischer exact tests. Kaplan–Meier survival analysis was used to construct survival curves. Mean time of death was compared using a one-way ANOVA with Tukey, LSD, and Bonferroni post-hoc analysis. Kruskal-Wallis tests were used to compare the colony forming units/gram of tissue. All analyses were conducted using IBM SPSS Statistics version 21.

**Figure 1:**
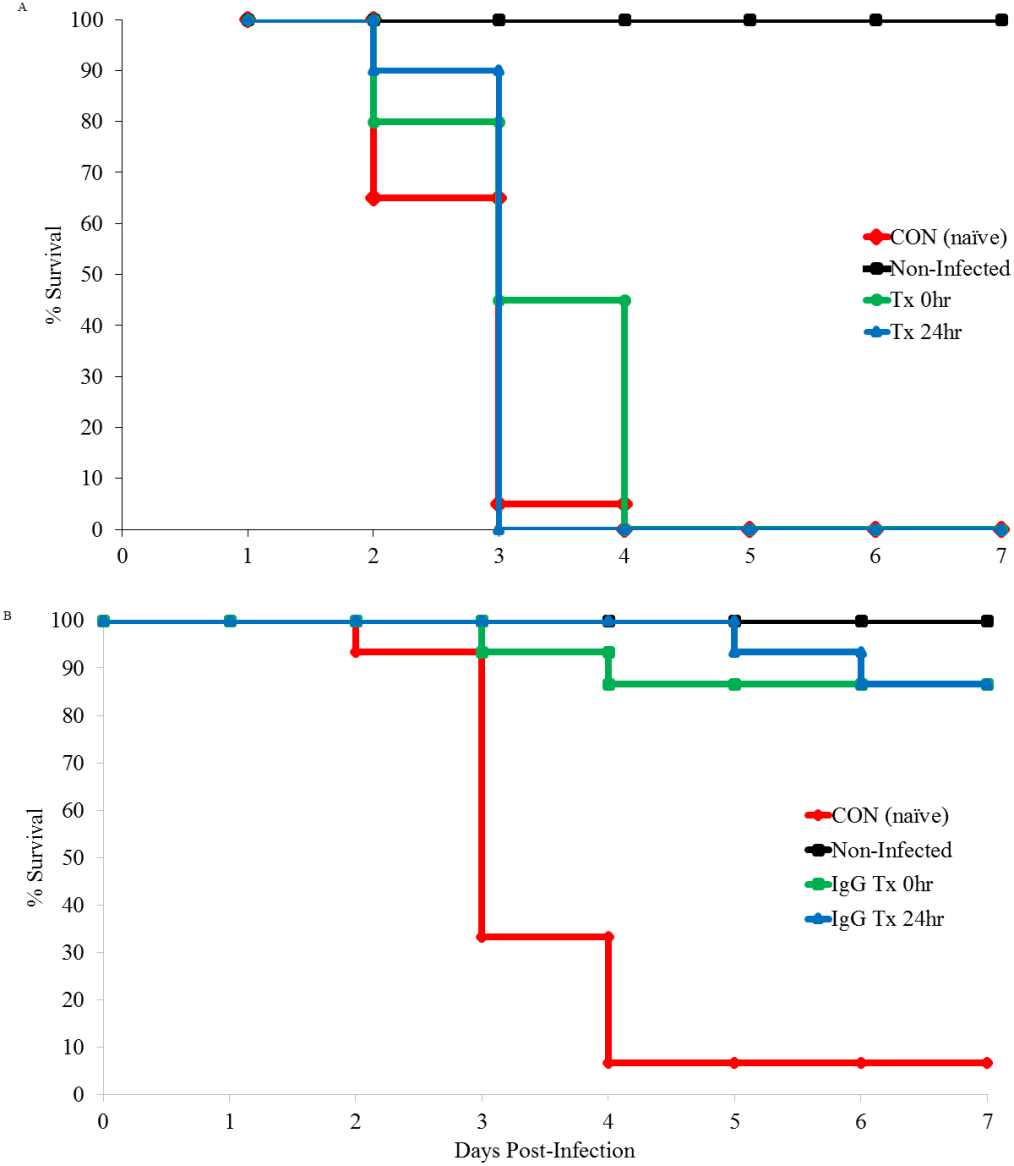
Kaplan Meier survival curve following IN challenge and treatment with immune plasma or purified immunoglobulins. (A) Mice were IN challenged with 1.5 × 10^4^ *B. anthracis* Sterne strain and treated with 0.8 ml of immune plasma at the time of inoculation (Tx 0 h, *n* = 20) or 24 h post-inoculation (Tx 24 h, *n* = 20). CON (naïve, *n* = 20) mice received the virulent challenge but were received 0.8 ml of naïve plasma at the time of inoculation. Non-infected mice served as treatment control mice and were sham inoculated with sterile PBS and treated with 0.8 ml of immune plasma at 0 h (*n* = 20). (B) Mice were IN challenged with 1.5 × 10^4^ *B. anthracis* Sterne strain and treated with 0.8 ml of purified immunoglobulins at the time of inoculation (IgG 0 h, *n* = 15) or 24-hours post-inoculation (IgG 24 h, *n* = 15). CON (naïve, *n* = 15) mice received the virulent challenge but were received 0.8 ml of CON immunoglobulins at the time of inoculation. Non-infected mice served as treatment control mice and were challenged with IN sterile PBS and treated with 0.8 ml of purified immunoglobulins at the time of inoculation (*n* = 15).

## Results

### Induction of anti-PA and neutralizing antibodies

Anti-PA IgG antibody titer responses were induced in each immunized horse. Geometric mean titers of 1:32,000 were achieved within two immunizations in 3 of 4 horses. Maximum anti-PA IgG titer induction was 1:128,000, though was not consistently maintained throughout the study period, Table 1. A consistent lethal toxin neutralizing antibody titer of 1:128 was induced following two immunizations in all horses and maintained throughout the study period. The maximum neutralizing titer observed was 1:256 for a single horse at one time point, Table 2.

### Survival of A/J mice following Sterne strain challenge

Figure 1 and Table 3 show the results of the Kaplan–Meier survival curve, cumulative survival, and mean to death of A/J mice following an IN challenge with 1.5 × 10^4^ spores and treated with immune plasma, purified immunoglobulins or placebo. Treatment with immune plasma at each time point resulted in a significantly prolonged mean time to death (*p* < 0.001), but did not significantly increase the number of mice (0/20 for 0 hr and 24 hr treatments) surviving to the end of the 7-day study period compared to controls (*p* = 1.000). When mice were administered 0.8 ml of purified immunoglobulins IP following IN challenge, 86% (13/15) survived to the end of trial when treated at the time of challenge and 80% (12/15) survived to the end of the trial when treated at 24 h post-challenge (*p* < 0.001, both treatment groups). In addition, treatment with purified immunoglobulins resulted in significantly prolonged survival time (*p* < 0.001, both treatment groups) when compared to mice treated with naïve plasma.

**Figure 2:**
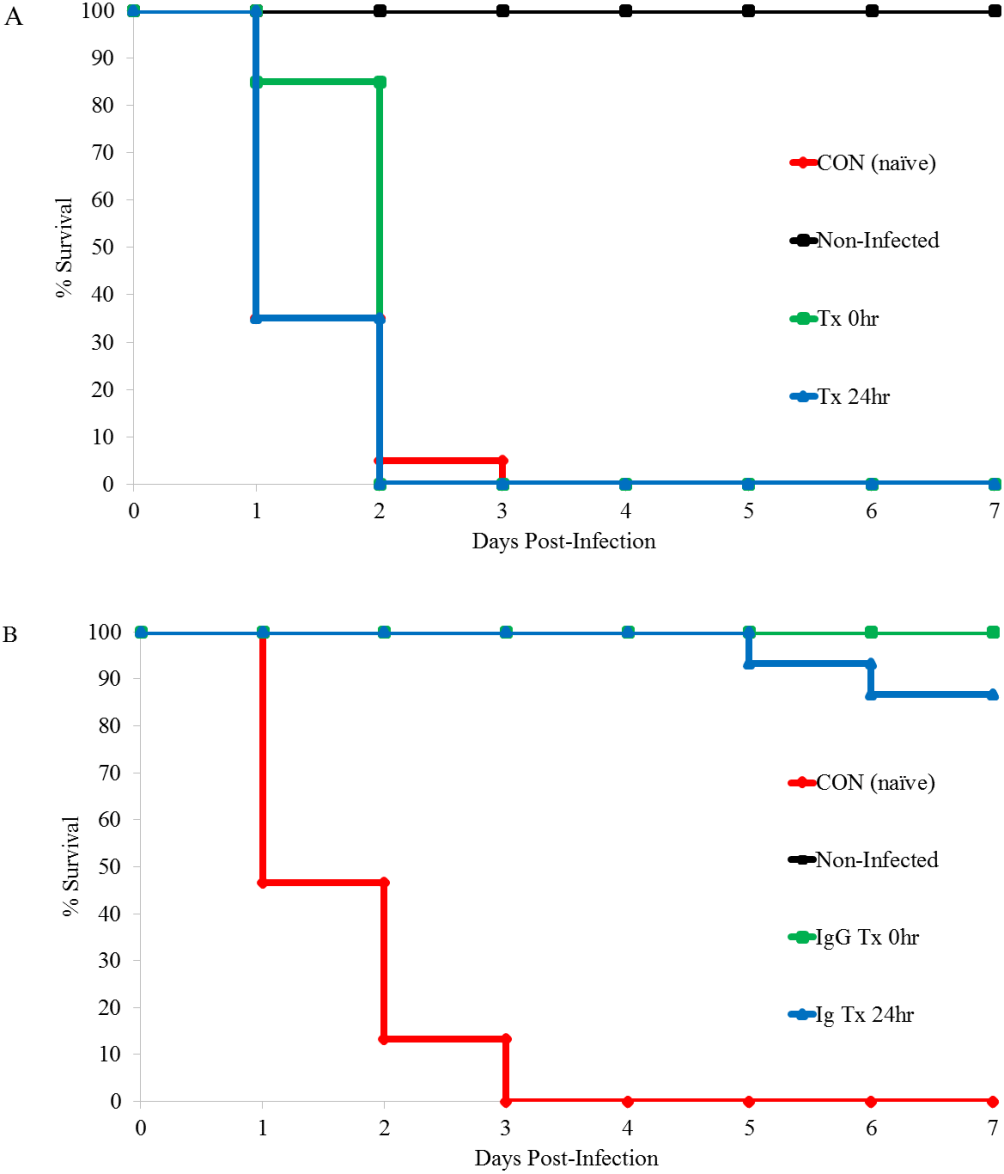
Kaplan Meier survival curve following SC challenge and treatment with immune plasma or purified immunoglobulin. (A) Mice were SC challenged with 2 × 10^3^ *B. anthracis* Sterne strain and treated with 0.8 ml of immune plasma or CON (placebo, *n* = 20) at 0 h (*n* = 20) or 24 h post-infection (*n* = 20). Non-Infected served as treatment control mice and were challenged with SC sterile PBS and treated with 0.8 ml of immune plasma at 0 h (*n* = 20). (B) Mice were SC challenged with 2.3 × 10^3^ *B. anthracis* Sterne strain and treated with 0.8 ml of purified immunoglobulins or CON immunoglobulins (placebo, *n* = 15) at 0 h (*n* = 15) or 24 h (*n* = 15) post-infection. Non-infected mice served as treatment control mice and were challenged with SC sterile PBS and treated with 0.8 ml of immune plasma at 0 h (*n* = 15).

Figure 2 and Table 4 reveal similar results for the SC challenge in that immune plasma treatments did not result in significantly more mice surviving to the end of the study period (*p* = 1.000, 0/20 for 0 hr and 24 hr treatment). Immune plasma treatment in SC challenged mice did lead to significantly longer mean time to death (*p* = 0.003) however, in this case for the time 0 h treatment group only. When SC challenged mice were treated with purified immunoglobulins, 100% (15/15) treated at time 0 h survived to the end of the study while 86% (13/15) treated 24 h following challenge survived. These survival rates were significantly higher (*p* < 0.001, both treatment groups) than challenge control mice. Moreover, treatment at both time periods resulted in significantly longer (*p* < 0.001) mean time to death compared to control mice. All treatments of naïve plasma, immune plasma and purified immunoglobulins were well tolerated by the non-challenged treatment control mice and no morbidity/mortality was recorded in these groups for any of the conducted trials.

**Table 3:** Cumulative survival and mean time to death following IN challenge. Mice were IN challenged with 1.5 × 10^4^ *B. anthracis* Sterne strain and treated with 0.8 ml of immune plasma, purified immunoglobulins, or CON plasma/immunoglobulins at 0 h or 24 h post-infection. Two independent trials were conducted, first using immune plasma as treatment, second using purified immunoglobulins. Group numbers: Plasma Tx 0 h *n* = 20, Plasma Tx 24 h *n* = 20, IgG 0 h *n* = 15, IgG 24 h *n* = 15, CON *n* = 20 first experiment, *n* = 15 s experiment, Non-infected treatment control *n* = 20 first experiment, *n* = 15 s experiment.

	Cumulative survival (survivors/total)			Mean time to death (Days)		
	0 h	24 h		0 h	24 h	
Immune Plasma (Tx groups)	0/20	0/20	*p* = 1.000	4.1	3.9	*p* < 0.001^a^
Purified Immunoglobulins (IgG groups)	14/15	12/15	*p* < 0.001^a^	6.5	6.8	*p* < 0.001^a^
CON (naïve)^b^	1/35	–		3.4	–	
Non-infected^b^	35/35	–		7.0	–	

**Notes.**

a *p* < 0.001 for both treatments compared to CON (placebo) group.

b Data presented represents the combined total of mice used in each independent trial (*n* = 20 for immune plasma trial, *n* = 15 for purified immunoglobulin trial).

**Table 4:** Cumulative survival and mean time to death following SC challenge. Mice were SC challenged with 2 × 10^3^ *B. anthracis* Sterne strain and treated with 0.8 ml of immune plasma, purified immunoglobulins, or naïve equine plasma at 0 h or 24 h post-infection. Group numbers: Plasma Tx 0 h *n* = 20, Plasma Tx 24 h *n* = 20, IgG 0 h *n* = 15, IgG 24 h *n* = 15, CON *n* = 20 first experiment, *n* = 15 s experiment, Non-infected treatment control *n* = 20 first experiment, *n* = 15 s experiment.

	Cumulative Survival (survivors/total)			Mean Time to Death (Days)		
	0 h	24 h		0 h	24 h	
Immune Plasma	0/20	0/20	*p* = 1.000	2.2	1.8	*p* = 0.003^b^
						*p* = 0.380^c^
Purified Immunoglobulins	14/15	13/15	*p* < 0.001^a^	7.0	6.8	*p* < 0.001^a^
CON (naïve)^d^	0/35	–		1.7	–	
Non-infected^d^	35/35	–		7.0	–	

**Notes.**

a *p* < 0.001 for both 0 hr and 24 hr treatment groups.

b *p* value for 0 hr immune plasma treatment.

c *p* value for 24 hr immune plasma treatment.

d Data presented represents the combined total of mice used in each independent trial (*n* = 20 for immune plasma trial, *n* = 15 for purified immunoglobulin trial).

### Bacterial concentrations

*B. anthracis* was successfully cultured from the lung, liver and spleen of infected mice in both challenge models. The tissues of non-infected control mice yielded no bacterial growth in any of the conducted trials. Although *B. anthracis* could be successfully isolated from the liver and spleen of mice following IN challenge, only lung tissue homogenates resulted in sufficiently high enough CFUs to accurately estimate bacterial loads. Treatment of IN challenged mice with immune plasma at either time point had no significant effect on the reduction in mean CFU/gram of lung tissue compared to non-treated challenge controls, Table 5. However, treatment of IN challenged mice with purified immunoglobulins at times 0 h and 24 h resulted in a 3-fold and 4-fold reduction in mean CFU/gram of lung tissue, respectively.

**Table 5:** Mean colony forming units (CFUs)/ gram of tissue. The lungs, liver and spleen were collected at necropsy from each mouse upon death. Each organ was weighed and homogenized then serially diluted two-fold into sterile saline. Each dilution was plated onto BHI agar and the quantification of *B. anthracis* vegetative cells or spores was determined by the Miles and Mirsa method. Values represent the mean CFU/gram for each treatment group and challenge, NG = no quantifiable growth, p values denote significance compared to challenge control in Kruskal-Wallis comparison of means.

Mean CFU/gram of tissue							
	IN challenge			SC challenge			
	Lung		Liver	Lung		Liver	
Immune Plasma 0 h Treatment (*n* = 20)	3.80 × 10^7^	*p* = 0.412	NG	1.25 × 10^5^	*p* = 0.844	7.4 × 10^4^	*p* = 0.233
Immune Plasma 24 h Treatment (*n* = 20)	4.90 × 10^7^	*p* = 0.634	NG	1.38 × 10^5^	*p* = 0.472	7.75 × 10^4^	*p* = 0.380
Purified Immunoglobulins 0 h Treatment (*n* = 15)	1.82 × 10^3^	*p* < 0.0001	NG	NG	*p* < 0.0001	NG	*p* < 0.0001
Purified Immunoglobulins 24 h Treatment (*n* = 15)	9.94 × 10^4^	*p* < 0.0001	NG	NG	*p* < 0.0001	1.11 × 10^3^	*p* < 0.0001
CON (placebo, *n* = 35)	7.57 × 10^7^		NG	1.15 × 10^5^		3.62 × 10^5^	
Non-infected (*n* = 35)	NG		NG	NG		NG	

Following SC challenge, culture of lung and liver homogenates, but not spleen homogenates, yielded sufficient CFUs for accurate bacterial load estimation. Treatment of SC challenged mice with immune plasma at both time points had no significant effect on the reduction in mean CFU/gram of lung or liver tissue when compared to non-treated challenge controls, Table 5. When mice were treated with purified immunoglobulins, lung and liver tissue of all surviving mice resulted in no bacterial growth (*p* < 0.0001 for each).

## Discussion

The results of this study demonstrate the protective capacity of purified immunoglobulins against *B. anthracis* harvested from horses immunized with the Sterne strain spore vaccine. We have quantified the activity of equine hyper-immune plasma against important *B. anthracis* antigens such PA and lethal toxin that have in several studies been implicated as the primary targets for immune protection (Ivins et al., 1992; Pohl et al., 2013; Turnbull et al., 1986; Wright et al., 2010). Moreover, in a lethal challenge mice were protected following passive transfer of purified IgG immunoglobulins at 0 h and 24-hour post-exposure. These findings satisfy the proof-of-concept objectives set forth and indicate that further development of an equine-derived immunotherapeutic against *B. anthracis* may be warranted.

The *B. anthracis* Sterne strain vaccine has been shown to induce a broad humoral response against all three toxin components as well as inducing innate responses and modulating macrophage activity (Chakrabarty et al., 2006; Ivins et al., 1986; Langel et al., 2014; Little & Knudson, 1986). This diverse immune response has been shown to be fully protective in both toxin challenges and fully virulent anthrax infections (Ivins et al., 1986; Jackson, Wright & Armstrong, 1957; Little & Knudson, 1986; Romanov, 1980; Welkos & Friedlander, 1988). In this study, the plasma harvested from Sterne strain vaccinated horses demonstrated a strong and rapid anti-PA and anti-lethal toxin antibody titer. Elsewhere these targets and quantitative methods have been shown to correlate with survival following experimental challenge (Taft & Weiss, 2008).

The use of A/J mice has proven a valuable challenge model for active and passive immunization studies with *B. anthracis* (Duong, Chiaraviglio & Kirby, 2006; Welkos, Keener & Gibbs, 1986). The A/J strain of mice is homozygous for *Naip5*^*Lgn*1−*s*^ which imparts reduced macrophage bactericidal activity and increased susceptibility to bacterial infection (Goossens, 2009). Welkos, Keener & Gibbs (1986) defined this strain’s susceptibility to *B. anthracis* Sterne strain by multiple routes of infection. This model provides a convenient example of a lethal challenge in that it greatly reduces the risk in handling an attenuated strain compared to fully virulent *B. anthracis* strains. This permits conducting such experiments in BSL 2 laboratory conditions. The limitation of the model is the potential for restricted relevance to fully virulent infections in larger animals and therefore extrapolation is precarious.

The treatments in this study were provided at the same time as the challenge or 24 h following challenge. It is unlikely that individuals exposed to *B. anthracis* would receive therapy at or near the time of infection, however treatment at 0 and 24 h post-infection provides a baseline to assess the protection of equine-derived hyperimmune plasma. Future therapeutic intervention studies that reflect the timing of infection and post-exposure prophylaxis are required. At this time, raxibacumab and AVIG are only approved as ancillary treatments alongside antimicrobial therapy and similar studies that evaluate the comparable effects of treatment with an equine-derived immunotherapeutic when combined with an antimicrobial are needed. The finding that treatment with purifiedIgG immunoglobulins reduced the bacterial infection load of the lungs or liver, but did not completely clear all of the spores or vegetative *B. anthracis,* indicates that a single treatment at either time point may not have been entirely protective if the study period had been extended beyond 7 days. This also suggest that perhaps additional immunoglobulin treatments or combination therapies such as concurrent antimicrobials would be beneficial.

The lack of survival in both routes of infection (IN and SC) in mice treated with immune plasma suggests that the titer of transferred antibodies was too low to sufficiently neutralize all circulating toxin and provide protection. One hypothesis for the poor protection of these mice is that the passive titers were rapidly consumed by a high toxin burden, which permitted continued progression of the disease. This hypothesis is supported by the evidence that treatment with immune plasma did prolong the mean time to death. Moreover, the survival of mice treated with purified immunoglobulins likely infers a higher treatment success resulting from the transfer of a much higher titer of protective antibodies. Another potential mechanism for the poor protection of the immune plasma is the increased non-specific clearance of the foreign proteins by the immune system. In this study, the titers of specific antibodies present in mouse serum post-transfusion, though valuable, were not determined nor were the kinetics of transferred antibody titers following immunoglobulin administration.

Testing both these hypotheses begins with determining the kinetics of passive titers in mouse serum over time in the anti-PA ELISA and toxin neutralization assays. It’s difficult to anticipate if the increased clearance of foreign protein would induce a measurable clinical effect, but all treatments were very well tolerated in non-challenged treatment control groups. Ideally, a correlation between the *in vivo* passive protection and TNA and ELISA titers would be made to define the dose of antibodies necessary to provide protection. The prolongation in mean to death suggests that multiple treatments of immune plasma may be necessary to establish full protection. Once the half-life of passive antibodies is determined then studies can be conducted to establish a dosing frequency necessary for successive treatments of immune plasma and/or a dose titration of purified immunoglobulins.

Mice that received immune plasma had similar bacterial concentrations (CFU/gram) upon death relative to CON treated mice, suggesting failure of the plasma to reduce bacterial concentrations. Treatment with purified immunoglobulins reduced the bacterial load in the lungs of IN challenged mice by fourfold, but did not eliminate the organisms. Oscherwitz et al. (2015) demonstrated that immunization of animals with spore antigens induced a protective antibody response that effectively limited germination. The passive treatments may have effectively conferred anti-spore antibodies and limited germination in treated mice. However, a longer period of observation may have allowed later germination of organisms and a delayed onset of disease.

In summary, though not exhaustive in its approach, this study establishes that an immune product with quantifiable anti-PA and lethal toxin neturalizing titers can be produced following vaccination of horses with the Sterne strain spore vaccine and can passively protect an alternate species from a lethal *B. anthracis* challenge. Future directions in developing equine-derived immunotherapeutics for anthrax include evaluation of thekinetics of passively transferred antibodies in the A/J mouse Sterne challenge model as well as evaluation of the protection in other more relevant animal challenge models and against more virulent strains. The plasma and immunoglobulin treatments administered in this study were unprocessed full-length immunoglobulins contained in raw or minimally purified plasma preparations. The Fc portion of the heavy and light chains are the most reactive segments of foreign immunoglobulins and typically these subunits are cleaved and removed following pepsin digestion and immunopurification in final therapeutic preparations. In the development of an approved equine-derived based immunotherapeutic, extensive post collection processing must be undertaken and evaluated after each step. This study outlines the first of these steps and demonstrates that equine-derived purified immunoglobulins might be a viable strategy for anthrax counter measures.

##  Supplemental Information

10.7717/peerj.3907/supp-1 Supplemental Information 1 Mouse clinical data Included are datasets used to calculate survival curves.

10.7717/peerj.3907/supp-2 Supplemental Information 2 Mouse clinical data Uncollated dataset transcribed from the clinical sheets.

10.7717/peerj.3907/supp-3 Supplemental Information 3 Tissue culture dataset Included are the CFUs for each animal and tissue type used to calculate the CFUs/gram tissue.

10.7717/peerj.3907/supp-4 Supplemental Information 4 Combined titer dataset Included are the collated titers over time for PA ELISA and toxin neutralization assay.

## Additional Information and Declarations

### Data Availability

The following information was supplied regarding data availability: The raw data has been supplied as a Supplemental Dataset.
